# Kinetic and Isotherm Study of As(III) Removal from Aqueous Solution by PET Track-Etched Membranes Loaded with Copper Microtubes

**DOI:** 10.3390/membranes11020116

**Published:** 2021-02-06

**Authors:** Alyona V. Russakova, Liliya Sh. Altynbaeva, Murat Barsbay, Dmitriy A. Zheltov, Maxim V. Zdorovets, Anastassiya A. Mashentseva

**Affiliations:** 1 The School of Information Technologies and Intelligent Systems, D.Serikbayev East Kazakhstan State Technical University, 070004 Ust-Kamenogorsk, Kazakhstan; Arussakova@gmail.com; 2 The Institute of Nuclear Physics of the Republic of Kazakhstan, 050032 Almaty, Kazakhstan; lilija310378@gmail.com (L.S.A.); zheltovda@gmail.com (D.A.Z.); mzdorovets@gmail.com (M.V.Z.); 3 Department of Chemistry, L.N. Gumilyov Eurasian National University, 010008 Nur-Sultan, Kazakhstan; 4 Department of Chemistry, Hacettepe University, 06800 Ankara, Turkey; mbarsbay@hacettepe.edu.tr; 5 Department of Intelligent Information Technologies, The Ural Federal University, 620002 Yekaterinburg, Russia; 6 Engineering Profile Laboratory, L.N. Gumilyov Eurasian National University, 010008 Nur-Sultan, Kazakhstan

**Keywords:** composite track-etched membranes, template synthesis, electroless plating, copper microtubes, arsenic (III) ions, adsorption kinetics

## Abstract

This paper reports on the synthesis and structure elucidation of track-etched membranes (TeMs) with electrolessly deposited copper microtubes (prepared in etched-only and oxidized polyethylene terephthalate (PET) TeMs), as well as on the comparative testing of arsenic (III) ion removal capacities through bath adsorption experiments. The structure and composition of composites were investigated by X-ray diffraction technique and scanning electron and atomic force microscopies. It was determined that adsorption followed pseudo-second-order kinetics, and the adsorption rate constants were calculated. A comparative study of the applicability of the adsorption models of Langmuir, Freundlich, and Dubinin–Radushkevich was carried out in order to describe the experimental isotherms of the prepared composite TeMs. The constants and parameters of all of the above equations were determined. By comparing the regression coefficients R^2^, it was shown that the Freundlich model describes the experimental data on the adsorption of arsenic through the studied samples better than others. Free energy of As(III) adsorption on the samples was determined using the Dubinin–Radushkevich isotherm model and was found to be 17.2 and 31.6 kJ/mol for Cu/PET and Cu/Ox_PET samples, respectively. The high $E_{Dr}$ value observed for the Cu/Ox_PET composite indicates that the interaction between the adsorbate and the composite is based on chemisorption.

## 1. Introduction

Increasing public awareness of the environment, concerns about depleting natural resources, and environmental disasters are driving interest in developing new methods and materials to treat contaminated media. Among a wide range of pollutants, heavy metal ions are one of the most serious ones as they are considered toxic even in trace amounts and are commonly present in various matrices, such as surface water, groundwater, sediments, and soils [1,2,3]. Arsenic is a widely distributed natural component of the earth’s crust. Its inorganic form is a highly toxic, clinically confirmed carcinogen and is the most significant chemical contaminant in drinking water globally [4]. The greatest threat to public health from arsenic originates from contaminated groundwater. In addition to pollution resulting from rapid and uncontrolled industrialization, inorganic arsenic is also naturally present at high levels in the groundwater of many countries, including the USA, China, India, Mexico, Argentina, Bangladesh, Hungary, and Chile [5].

The most widely applied treatment scheme for the removal of arsenic contamination is adsorption [3]. However, disposal of the adsorbent (for example, in landfills) only transfers the problem to another medium. Therefore, scientific and industrial efforts are focused on the development of new materials that will effectively capture the highest amount of contaminants with the least amount of adsorbent [3]. For this very reason, nanomaterials stand out as suitable materials that can meet this need due to their high surface areas and specificity. In addition to their adsorptive capacity, nanoparticles (NPs) may be excellent candidates for water treatment [6,7,8] applications due to their high reactivity, for example, through catalytic reactions [9]. In many previous studies, various NPs have been examined as adsorption media in arsenic removal [10,11,12,13]. Among them, the most commonly used are iron oxides/hydroxides [10,14]. It is well known that oxides of polyvalent metals, such as Fe(III), Ti(IV), Cu(II), and Al(III), exhibit ligand sorption properties through the formation of inner-sphere complexes [15,16,17]. Therefore, oxides of metals other than iron have also been studied widely for the removal of inorganic arsenic species. Among these metal oxides, TiO_2_, CeO_2_, Al_2_O_3_, ZrO_2_, CaO_2_, and CuO are promising due to their high adsorption capacities [10,11,12,18,19].

Although these NPs are effective in the selective adsorption of arsenic, their applications suffer from the formation of a large amount of sludge containing a substantial concentration of arsenic [10]. For this reason, employing NPs in heterogeneous systems, particularly in a nanoporous membrane with a large surface area, such as track-etched membranes (TeMs), offers ease of use along with high capacity and creates less burden on the environment, which is easier to deal with after treatment. When a polymer membrane is irradiated by swift heavy ions, ions penetrating through the solid induce continuous trails of excitations and ionizations in their pathways, leading to the formation of latent tracks. These latent tracks are revealed when selectively etched in a highly oxidizing solution, leading to nanoporous TeMs. Nowadays, TeMs are used as filtration membranes for water purification [20,21] or effective adsorbents and are commercially available. Combining the inherent advantages of TeMs and metal nanostructures provides composite membranes that are excellent candidates in a variety of promising material science applications, such as biotechnology [22,23], catalysis [24,25,26,27,28], medicine [29,30], sensors [21,31], and radiation resistive and magnetic materials [32,33]. In our previous study, we created Cu/CuO in the nanochannels and surface of poly(ethylene terephthalate) (PET) TeMs by electroless template-assisted deposition of copper and subsequent low-temperature annealing process. The resulting composite membrane was successfully evaluated in cross-flow mode as an adsorbent for the removal of As(III) from wastewater samples [34]. In the present work, we excluded the thermal annellation step from the procedure, thus demonstrating that efficient membranes for As(III) removal can be developed with a simpler and one-step synthesis method. Furthermore, unlike in our previous study, we evaluated the removal of As(III) not only by using track-etched PET but also by employing oxidized PET TeM as the template. As a result, we recommend oxidation as a step that can be considered in the preparation of similar membranes since a higher arsenic removal efficiency was obtained using the oxidized PET TeM. Finally, a detailed study was carried out to elucidate the kinetics and adsorption mechanism of As(III) on the surface of composite TeMs with embedded copper microtubes (MTs).

## 2. Materials and Methods

### 2.1. Materials

Copper(II) sulfate pentahydrate, sodium potassium tartrate, and palladium chloride were purchased from Sigma-Aldrich. All chemical reactants were of the analytical or reagent grade purity and used without further purification. The certified reference solution at a concentration of 0.1 g/L As(III) was purchased from Ecroskhim (Russia). The water used in all the experiments was purified using a D-301 water purification system (Akvilon, Russia) with a resistivity of 18.2 MΩ/cm.

To obtain polymer templates, a PET Hostaphan^®^ RNK film (film thickness is 12.0 microns) was irradiated by ^84^Kr^15+^ ions with 1.75 MeV/nucleon energy and 4 × 10^7^ ion/cm^2^ fluency (Cyclotron DC-60, Institute of Nuclear Physics of Kazakhstan) and then etched in 2.2 M NaOH.

### 2.2. Composite TeMs Synthesis

All composites were prepared using an oxidized or non-oxidized (only etched) PET template with 430 ± 10 nm pore size. Cu/Ox_PET composites were prepared using a pre-oxidized PET template. The oxidized PET (Ox_PET) was obtained by exposing pristine PET TeMs to a 500 mM H_2_O_2_ solution at pH 3 for 180 min under UV irradiation (190 W at 254 nm). After oxidation, the samples were washed twice with deionized water and air-dried at room temperature for 5 h [35]. Cu/PET composite was prepared using non-oxidized PET TeMs. Electroless plating of copper was accomplished by following three steps:

− Sensitization step: PET template was immersed in 50 g/L SnCl_2_ and 60 mL/L 37% HCl solution for 6 min and rinsed thoroughly in hot water for 10 min.
− Activation step: the sensitized membrane was immersed in a solution of 0.1 g/L of PdCl_2_ and 10 mL/L of HCl for 6 min and then air-dried.
− Deposition step: activated polymer template was immersed in a thermostated deposition solution for 40 min at a temperature of 283 K (KNaC_4_H_4_O_6_·4H_2_O, 18 g/L; CuSO_4_·5H_2_O, 5 g/L; NaOH, 7 g/L; formaldehyde, 0.13 M), pH 12.45 [36].

### 2.3. Characterization of PET Template and Composites

Fourier-transform infrared spectroscopy (FTIR) spectra were recorded using a Cary 600 Series FTIR spectrometer (Agilent Technologies, Santa Clara, CA, USA) with a single reflection diamond attenuated total reflectance (ATR) accessory (PIKE Technologies, Madison WI, United States of America). Measurements were taken in the range from 400 to 4000 cm^−1^. All spectra (32 scans at 2.0 cm^−1^ resolution and rationed to the appropriate background spectra) were recorded at room temperature.

XPS measurements were carried out using a Thermo Scientific K-Alpha spectrometer (Waltham, MA, USA) with a monochromatized Al Kα X-ray source (1486.6 eV photons) at a constant dwell time of 100 ms and pass energy of 30 eV with steps of 0.1 and 200 eV for core-level and survey scan spectra, respectively. The pressure in the analysis chamber was maintained at 2 × 10^−9^ Torr or lower. The binding energy (BE) values were referred to the C1s peak at 284.7 eV. Processing of data was carried out using the Avantage software.

The change in the concentration of carboxyl groups on the surface of the PET template after oxidative pretreatment was investigated based on the complexation between toluidine blue dye and carboxyl groups according to the procedure described elsewhere [37]. The calculations were based on the assumption that 1 M of dye is complexed with 1 M of the carboxyl group and expressed in nM/cm^2^.

The composites produced were characterized with various techniques. Scanning electron microscopy (SEM) images were taken using a JEOL JFC-7500F microscope (Tokyo, Japan). Prior to the SEM analysis, a 15 nm layer of gold was sputtered onto the membranes. In order to analyze the MTs filling the channels of PET, the PET template was dissolved in a mixture of 1,1,1,3,3,3-hexafluoro-2-propanol and chloroform. Then, the released copper MTs were analyzed by SEM. The chemical composition and morphology of the released copper MTs do not change, as discussed in our previous study [38]. To obtain high-quality cross-sectional images of the composite films, the composite membrane was irradiated by a UV lamp for 10 days from each side. The elemental composition of the composites was studied by a Hitachi TM3030 SEM (Hitachi Ltd., Chiyoda, Tokyo, Japan) equipped with a Bruker XFlash MIN SVE (Bruker, Karlsruhe, Germany) microanalysis system at an accelerating voltage of 15 kV. The specific surface area was determined from the N_2_ adsorption isotherm using the single-point BET method by a 3H-2000PS1 GVD3 pore size and surface analyzer (Xiamen Lith Machine Ltd., Guangzhou, China) at 77 K at a relative pressure range of 0.05 to 0.25. Prior to measurements, the samples were degassed at 373 K under vacuum for 12 h, and all measurements were repeated in triplicate.

The pore size of the pristine template and the structural parameters of the composites obtained were determined by porometry using the Hagen–Poiseuille equation [39]. X-ray diffraction (XRD) patterns were obtained on a D8 Advance diffractometer (Bruker, Karlsruhe, Germany) to study the crystalline structure of the samples. X-ray was generated at 25 mA and 40 kV, and the scanning position ranged from 30° to 90° 2(θ). The average crystallite size was determined using the Scherrer equation [40]. The surface morphology of the composite membranes was studied by a scanning probe microscope (SmartSPM-1000, NT-MDT, Novato, CA, United States of America) in semicontact mode using an NSG10 (TipsNano, Tallinn, Estonia) rectangular-shaped silicon cantilever (length, 95 ± 5 μm; width, 30 ± 5 μm; thickness, 1.5–2.5 μm; probe tip radius, 10 nm; resonance frequency, 200 kHz). Initial scanning of a 10 × 10 μm^2^ sample was performed at a speed of 5.0 μm/s. The average roughness was calculated from a 3 × 3 μm^2^ scanning area. The data obtained were processed and analyzed by using the IAPRO-3.2.2 software.

The amount of copper deposited was determined gravimetrically based on the difference in the weights of the composite before and after plating with an accuracy of 0.1 mg (AS 220.R2, Radwag, Radom, Poland) and expressed in units of mg/cm^2^.

### 2.4. Bath Absorption Experiments

All experiments conducted to determine the As(III) adsorption performance of composite TeMs and pristine templates were carried out using batch equilibrium techniques. Feed As(III) solution (10 ppm, pH 4.0) was prepared by diluting the certified As(III) reference solution (0.1 g/L, Ecroskhim, Russia). Adsorption kinetics were studied at an As(III) concentration of 50 μg/L (pH 4.0). Disposable plastic vials (Isolab, Eschau, Germany) containing 15.0 mL of solution and 2 × 2 cm of composite adsorbate were shaken (100 rpm, IKA KS 3000 IS control, (IKA, Konigswinter, Germany) at room temperature for different times between 15 min and 10 h. Each experiment was repeated in triplicate. The concentration of As(III) in aliquots was determined by ICP–MS (Thermo Fisher Scientific, XSeries 2, Bremen, Germany). The adsorbed amount of As(III) was calculated using Equation (1) [41,42,43]:

(1) $$Q_{e}=\frac{\left(C_{0}-C_{e}\right)\times V}{m}$$

where *Q_e_* is the amount of As(III) adsorbed by the unit mass of copper (mg/g), *C*_0_ is the feed concentration (mg/L), *C_e_* is the concentration of As(III) in aliquots (mg/L), *V* is the volume of the solution (L), and *m* is the amount of Cu loaded on the membrane used (g). In the case where the pristine template was tested, the weight of PET TeM was used in *m* (g).

The effect of pH on As(III) adsorption was studied in the pH range of 3 to 10. Other parameters were kept constant (initial As(III) concentration: 50 ppm; adsorbent dose: 2 × 2 cm^2^; contact time: 420 min). The pH of the solution was adjusted dropwise with 1.0 N HCl_(aq)_ and 1.0 N NaOH_(aq)_. The pH was measured using a digital pH meter, HANNA HI2020-02 (HANNA Instruments, Smithfield, United States of America). All experiments were performed in triplicate.

## 3. Results and Discussions

### 3.1. Characterization of the Composite Membranes

The development of new methodologies for the synthesis of functional materials aimed at obtaining samples with an improved structure and characteristics is one of the promising areas of materials science. As shown in our previous studies, the concentration of carboxyl groups directly affects the degree of sensitization; thus the oxidative pretreatment results in an increase in the catalytic activity of the composite membrane compared with a sample synthesized in “etched-only” PET TeM [35,44]. In this study, higher efficiency properties obtained with pretreated templates were utilized in As(III) sorption.

Both types of PET templates used (“etched-only” and “oxidized”) were studied by XPS technique. High-resolution C1s and O1s spectra of oxidized and etched-only PET templates are shown in Figure S1 of the Supplementary Material. The C1s spectrum is characterized by three peaks: C-C/C-H groups located at the binding energy of ~284.6 eV, C-OH/C-O-C groups at ~286.2 eV, and –COOH groups at ~288.6 eV. It is clear from the C1s spectra that the oxygenated C content is higher in the oxidized sample compared with the etched-only membrane. High-resolution O1s spectra have two peaks located at ~531.5 eV (O=C) and ~533.8 eV (O-C) [37]. After oxidation, there is a relative increase in the amount of O=C species compared with oxygen atoms attached to C atom with a single covalent bond (O-C). As a result of the analysis of the surface with XPS, it is clear that after the oxidative treatment of the PET template in the H_2_O_2_/UV system, the oxygen content increases compared with the “etched-only” PET template.

The terminal carboxyl group concentration ([–COOH]) was determined by the toluidine blue dye assay and was calculated as 6.4 ± 0.5 and 10.8 ± 0.2 nM/cm^2^ for the pristine and oxidized PET templates, respectively. The significant increase in the –COOH amount was also confirmed by the FTIR data presented in Figure S2 of the Supplementary Material. The main difference was observed at around 1715 cm^−1^ corresponding to the stretching vibrations of the C=O groups. After oxidation, the intensity of C=O peak increases due to the increasing concentration of polar carboxyl groups.

The electroless deposition of copper has been studied extensively, and a wide variety of plating solutions and various additives have previously been used in order to obtain thin copper layers or nanostructures [45,46,47,48]. As a result of the method we apply, the wall thickness and inner diameter obtained by SEM, specific surface area determined by BET analysis, and gravimetrically calculated amount of copper loaded to the sorbent are presented in Table 1. When comparing the amount of copper loaded, it is noted that more copper deposition occurs when the oxidized PET template is used due to the increase in the functional carboxyl groups. Furthermore, the results in this table clearly show that the specific surface area of the composite prepared using the oxidized PET is significantly higher than that obtained using the etched-only template. These results are in line with the atomic force microscopy (AFM) results that will be presented later. Both increased copper loading and surface area are associated with high As(III) adsorption capacity, which is obtained in the presence of oxidized PET and will be discussed in the following sections.

**Figure 1:**
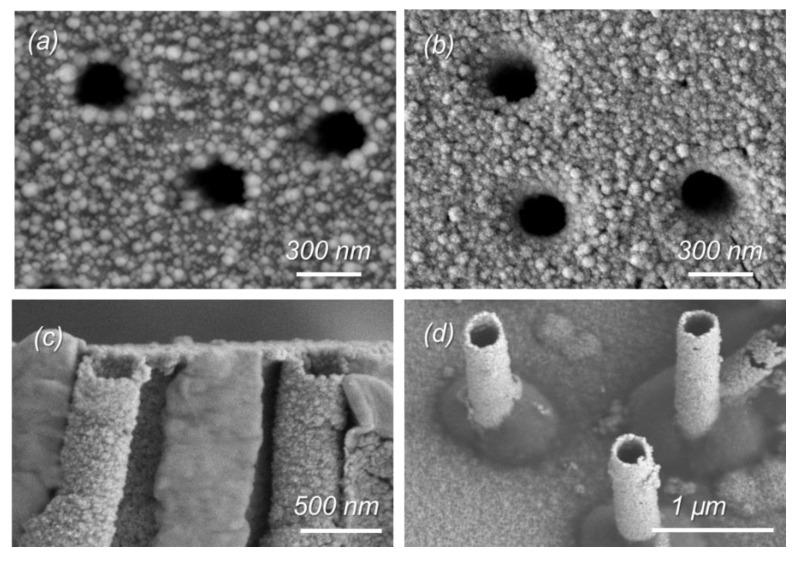
Scanning electron microscopy (SEM) images of the composite membranes of Cu/PET (**a**) and Cu/Ox_PET (**b**). SEM images of corresponding microtubes (MTs) released after the dissolution of the PET template (**c**,**d**).

As can be seen from the SEM images shown in Figure 1, all the samples studied are porous and homogeneously covered with copper. From the SEM images of the released MTs (Figure 1c–d), it is clear that the electroless plating of copper allows for synthesizing hollow tubular MTs with an outer diameter equal to the pore diameter of the pristine polymer template.

The chemical composition of the composite membranes was studied further by energy dispersive X-ray analysis (EDX). EDX spectra and element percentages for Cu/PET and Cu/Ox_PET samples are presented in Figure S3 of the Supplementary Material. Strong signals from the C and O elements can be attributed to the PET template. The absence of any impurity is clear in the spectra. Additionally, in accordance with other analysis results, the amounts of the Cu and O elements in the oxidized composite structure were higher.

**Figure 2:**
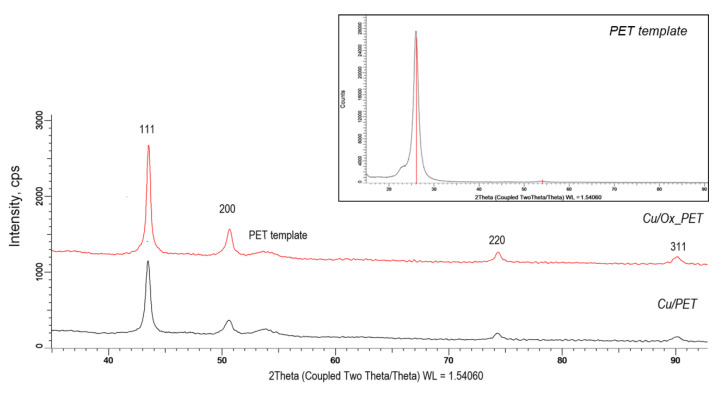
X-ray diffraction (XRD) patterns of the studied composite TeMs. The inset shows the XRD pattern of the PET template.

To evaluate the crystal structure and phase contents, XRD analysis of composite TeMs was carried out. As can be seen from the XRD patterns of composites (Figure 2), four peaks (111, 200, 220, and 311) can be assigned for Cu MT arrays, which possess a polycrystalline structure with a face-centered cubic (fcc) phase without the presence of oxide compounds in the structure. An additional broad peak in the region from 53° to 56° relates to the amorphous structure of the PET template, as can be seen from the XRD pattern of the pristine PET presented in the inset of Figure 2. The location and intensity of the peaks in the XRD diffractograms, their corresponding interplane distances, the full width at half maximum (FWHM) values, and the unit cell parameters and average crystallite sizes calculated for the studied composites are summarized in Table 2.

According to X-ray diffraction data, the unit cell is characterized by a cubic syngony (Fm3m) with a cell parameter of a = 3.604, slightly different from the reference value (a = 3.6150, PDF #040836). A small deviation of the reference value of the unit cell parameter and the sample value can be explained by microstresses arising in the structure during synthesis process. When the lines on the diffractogram were approximated by the necessary number of symmetric pseudo-Voigt functions, the width of the registered lines at half of their height (FWHM) was measured, which allowed us to estimate the degree of perfection of the crystal structure and the degree of crystallinity (DC). For the Cu/PET and Cu/Ox_PET samples, the DC values were calculated as a 43.8% and 52.5%, respectively.

### 3.2. Sorption Kinetics of As(III) on Composite TeMs

**Figure 3:**
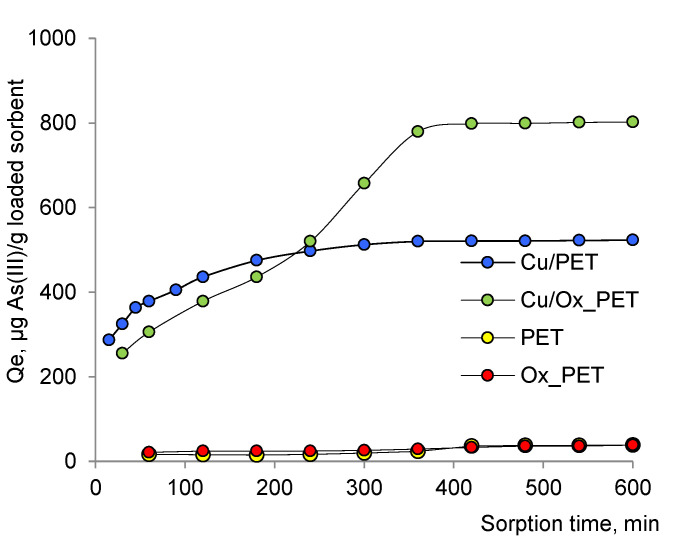
Effect of contact time on the sorption of As(III) (50 ppm) by the composite TeMs.

Adsorption is a time-dependent process and is affected by physical/chemical interactions between the adsorbent and the adsorbate. Studying adsorption kinetics is very important to understand the adsorption mechanism of new adsorbents. To investigate the adsorption kinetics of As(III) on composite TeMs, we used three kinetic models, namely, Elovich, pseudo-first-order, and pseudo-second-order models [49,50,51,52]. Figure 3 shows the amount of arsenic(III) adsorbed by composite TeMs with deposited metallic copper microtubes depending on the adsorption time. In order to exclude the effect of the PET template on the sorption activity of the prepared composites, etched-only (pristine) and oxidized PET species were also examined in As(III) sorption along with both types of composites. As can be seen in Figure 3, the PET template has a very low arsenic sorption capacity both when oxidized and only-etched, and the equilibrium sorption capacities (*Q_e_*) are 33.2 and 36.3 μg As(III)/g for the etched-only and oxidized PET templates, respectively.

It is clear from Figure 3 that when oxidized PET is used as the template, a much higher equilibrium sorption capacity (*Q_e_*) is achieved compared with what is obtained in the case of etched-only PET. While *Q_e_* was found as 802 μg As(III)/g for Cu/Ox_PET, it was 521 μg As(III)/g when un-oxidized PET was applied as a template. Since the PET template alone does not have much effect on adsorption, this significant increase is attributed to the increase in the amount of copper loaded to the membrane and the specific surface area (Table 1). Although the time required to reach the equilibrium sorption is slightly longer in oxidized PET (360 min) compared with the pristine counterpart (300 min), this is far from a problem given the high amount of equilibrium adsorption obtained.

**Figure 4:**
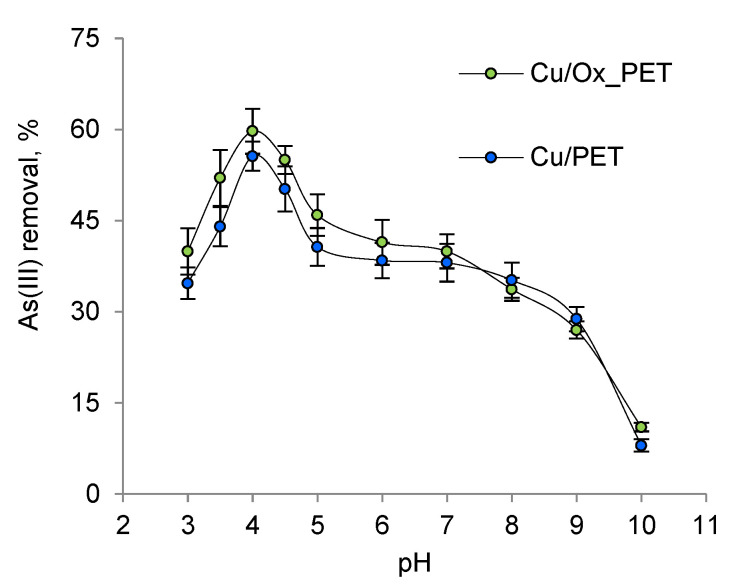
Removal of arsenic as a function of solution pH (As(III) concentration: 50 ppm; composite TeMs: 2 × 2 cm^2^; contact time: 420 min).

The relative distribution of As(III) species and the ionization of functional groups on the adsorbent surface depend on the pH of the solution [53,54]. When the effect of pH on As(III) sorption was examined, it was found that the maximum sorption capacity was obtained at pH 4 (Figure 4).

At pH > 9, the membranes lost their integrity due to the destruction of the PET template and were unable to function. As the pH increased from 3 to 4, the adsorption efficiency increased from 40% to 60%. As(III) removal decreased dramatically at pH > 4.0. The results show that the interactions between the composite absorbent and As(III) are highest at pH 4, which is related to both the surface charge of the absorbent and the charge and structure of As(III) species at this pH [55].

The pseudo-first-order model proposed by Lagergren [56] as the earliest adsorption kinetic model is used to describe the adsorption behavior of solid adsorbents in liquid media. The differential form of the pseudo-first-order model is given by Equation (2) [56]:

(2) $$\frac{dq_{t}}{d_{t}}=k_{1}\left(q_{e}-q_{t}\right)$$

**Figure 5:**
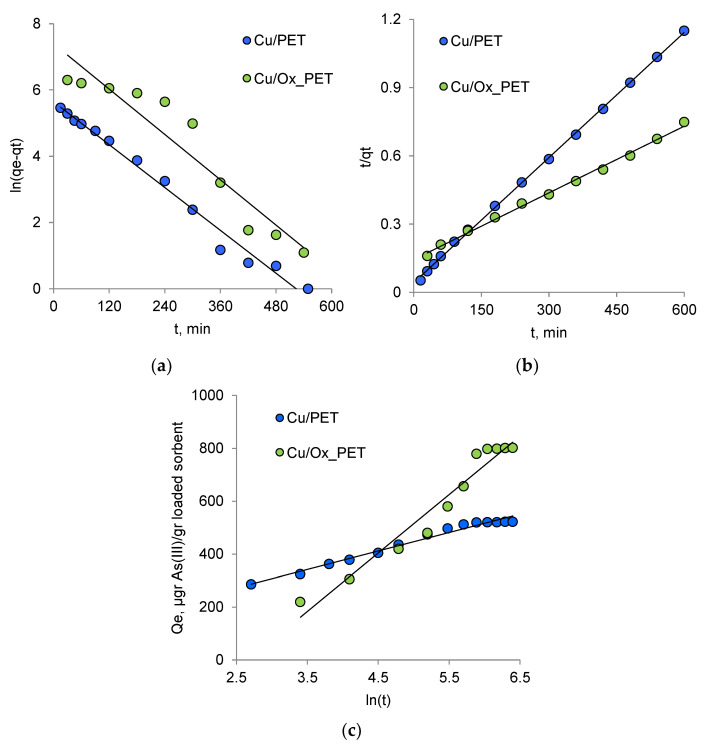
Kinetics of As(III) sorption by composite TeMs according to the pseudo-first-order (**a**) and pseudo-second-order (**b**) and Elovich (**c**) kinetic models.

Equation (2) can be expressed as follows after integration to obtain a linear form:

(3) $$\ln\left(q_{e}-q_{t}\right)=ln\;q_{e}-k_{1}t$$

where $q_{t}$ is the adsorption capacity at time t, mg/g, and $k_{1}$ is the first-order reaction rate constant. As shown in Figure 5a and listed in Table 3, the values of $k_{1}$ and $q_{e}$ and the coefficient of determination, R^2^, are determined by the linear graph dependency. For the pseudo-first-order kinetic model, the lower value of R^2^ indicates that the adsorption kinetics do not match the pseudo-first-order reaction kinetic model.

When the concentration of adsorbate ions reaching the adsorbent functional groups is extremely low compared with the active centers, the first-order kinetic model can be applied to the initial stage of the adsorption process [57]. In this case, the number of active centers involved in the adsorption changes slightly over time, and the adsorption process can be reduced mathematically by including the concentration of the functional groups of the adsorbent in the reaction rate constant. In the latter stage of the adsorption process, the adsorption rate is affected by the concentration of the two components, so the order of the adsorption becomes two.

If the process is defined by the pseudo-second-order model (Equation (4)), the interaction between the adsorbate and the functional group of the adsorbent is strictly stoichiometric; that is, a metal ion occupies an adsorption site [58].

In general, this model is similar to the pseudo-first-order kinetic model, but it can be used to describe the entire adsorption process [59].

(4) $$\frac{dq_{t}}{t}=k_{2}{\left(q_{e}-q_{t}\right)}^{2}$$

By integrating *t* from 0 to *t* and $q_{0}$ from 0 to $q_{t},$ we obtain

(5) $$q_{t}=\frac{k_{2}q_{e}^{2}t^{}}{1+k_{2}q_{e}^{}t}$$

In linear form, the equation becomes

(6) $$\frac{t}{q_{t}}=\frac{1}{k_{2}q_{e}^{2}}+\frac{t}{q_{e}}$$

where $k_{2}$ is the pseudo-second-order rate constant of the adsorption, $\frac{g}{\mathrm{mg}\times \min}$, and $q_{e}$ is the equilibrium sorption amount, mg/g. These two values and the corresponding linear regression correlation coefficient R^2^ are calculated from the linear graph shown in Figure 5b, and the results are presented in Table 3. The calculated $q_{e}$ values of Cu/PET and Cu/Ox_PET are 0.545 and 1.0 mg/g, respectively, which are very consistent with the experimental data under the pseudo-second-order kinetics.

The pseudo-second-order equation also covers the intermolecular interactions of the adsorbate. The Elovich model described in Equation (7) takes into account the contribution of the adsorption process and the desorption phenomena to material extraction kinetics, which will have a significant impact when the adsorption is close to the equilibrium state [59].

(7) $$q_{t}=\frac{1}{\beta }\left(\ln\alpha \beta \right)+\frac{1}{\beta }\ln t$$

where $q_{t}$ is the amount of adsorbate at time t, μg/g; α is the initial rate of the adsorption process, mg/g × min; and β is the desorption constant (g·mmol^−1^). The kinetic parameters of this model are calculated from the linear dependence of $q_{t}$ on ln(t) (Figure 5c), and the results are given in Table 3. Compared with β, the higher-value α indicates that arsenic absorption, rather than its desorption, is dominant [59]. As can be seen in Table 3, the α value for Cu/Ox_PET is much higher than in the case where the non-oxidized PET template is used. The results presented in Figure 3 confirm that the PET template contributes to the adsorption process. The lower β value obtained in the case of the oxidized template states that the desorption of As(III) occurs at a lower amount, indicating favorable interaction between the adsorbate and the adsorbent through the oxidized species. When these two parameters, α and β, are evaluated together, the high equilibrium sorption obtained from the use of oxidized PET becomes quite expected.

The value of the correlation coefficient R^2^ shows that the pseudo-second-order model best describes the adsorption of arsenic(III) by both composite adsorbents. The linear dependence in Figure 5b over the entire time interval is obvious. The applicability of the pseudo-second-order kinetic model to both composite sorbents leads to the conclusion that chemisorption is the rate-determining step of the process, and the effect of the diffusion stage is insignificant [49,51].

**Figure 6:**
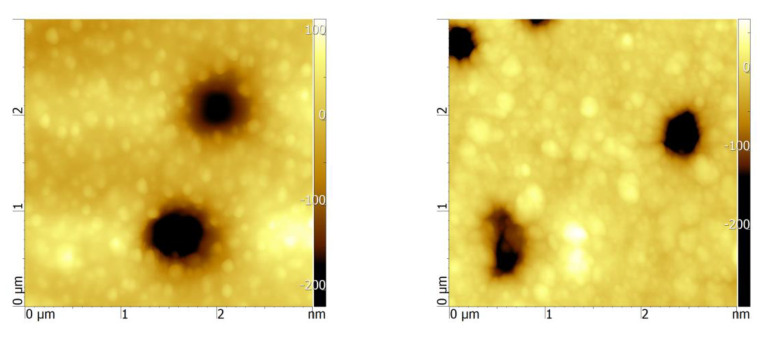

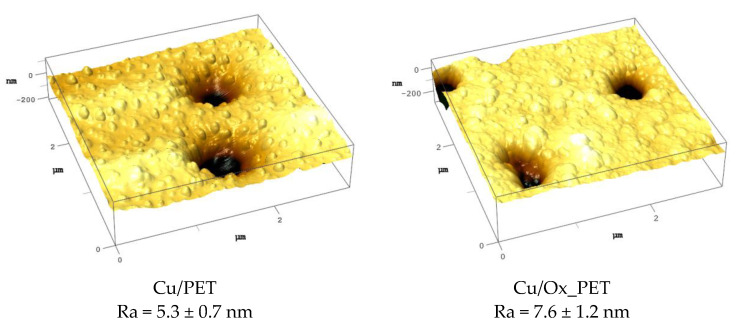
Atomic force microscopy (AFM) images of the surface of composite adsorbents. The scanning area was 3 × 3 μm^2^.

As can be seen in Table 1, the specific surface area and the amount of copper loaded are higher in the case of Cu/Ox_PET. The oxidation process is expected to increase surface porosity, and this morphological difference also positively contributes to the adsorption process. To confirm this expectation, we performed atomic force microscopy (Figure 6) and found that the roughness (Ra) of the composite obtained when using the oxidized PET template was 30% higher compared with the use of its untreated counterpart. In addition to all these, the Cu/Ox_PET composite has a smaller crystallite size (15.0 ± 2.8 nm) compared with Cu/PET (18.0 ± 2.7 nm), as was shown earlier [35]. Therefore, we think that smaller copper crystallites also contribute to increased adsorption performance with their increased surface areas.

### 3.3. Investigation of As(III) Adsorption Mechanism

The presence of toxic pollutants in the aquatic environment and the subsequent development of removal methods have promoted the rapid development of new adsorbents. It is very important to understand the adsorption process correctly and to reveal the adsorption equilibrium clearly. Correct understanding and interpretation of adsorption isotherms are essential for the improvement of adsorption pathways and an efficient sorption system design [12,60]. The equilibrium in the adsorption system depends on the nature of the interactions between the adsorbent and the adsorbate. Well-known adsorption models—Langmuir, Freundlich, and Dubinin–Radushkevich (DR)—describe these interactions in different ways [42,61]. Therefore, the purpose of this section is to clarify the applicability of these models in interpreting the experimental data for the adsorption of As(III) on composite TeMs with embedded copper MTs and to select the model that best describes the adsorption process.

#### 3.3.1. Langmuir Isotherm

According to the Langmuir adsorption theory, adsorption is local; it occurs on the active centers, and the active centers are equivalent. Each active site retains only one molecule, and adsorption saturation occurs when the active sites are filled. The adsorbed molecules do not interact with each other, and after a while, they are desorbed, thus achieving a dynamic equilibrium [60,62]. For reactions occurring in solution, the linear form of the Langmuir adsorption isotherm equation, derived on the basis of the molecular kinetic theory and concepts of the monomolecular nature of the adsorption process, can be presented in the following form [51]:

(8) $$\frac{C_{e}}{q_{e}}=\frac{C_{e}}{Q_{0}}+\frac{1}{Q_{0}b}$$

where *b* is a constant related to the energy of adsorption (Langmuir constant); *C_e_* is the equilibrium concentration of the adsorbate, mg/L; $q_{e}$ is the amount of arsenic adsorbed per gram of the adsorbent at equilibrium (mg/g); and $Q_{0}$ is the maximum monolayer coverage capacity (mg/g), which characterizes the amount of adsorbate that can be absorbed by a unit mass of the adsorbent to form a monolayer on the surface.

**Figure 7:**
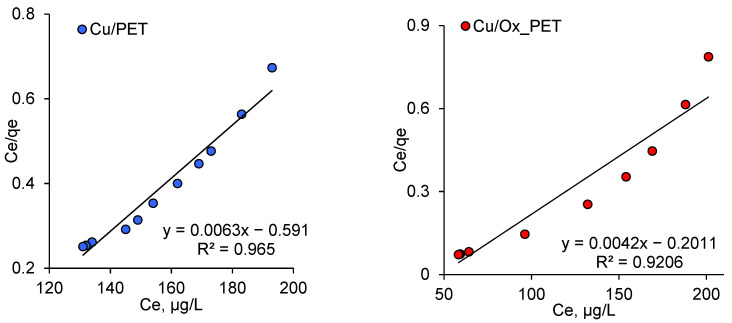
Langmuir adsorption isotherms of As(III) adsorption on composite TeMs.

Both parameters *b* and $Q_{0}$ are characteristic values that reflect the basic properties of the “adsorbent–adsorbate” pair. The linear plot of $C_{e}\;\mathrm{vs}.\;C_{e}$/$q_{e}$ dependence is shown in Figure 7. The values $Q_{0}$ and *b* were calculated from the slope and intersection of straight lines, respectively, and the results are presented in Table 4. As can be seen in Table 4, a higher $Q_{0}$ value was attained when using oxidized PET compared with its pristine counterpart, which indicates a higher sorption potential for Cu/Ox_PET and is consistent with previous discussions.

#### 3.3.2. The Isotherm of Freundlich

The Freundlich isotherm model (9) is used to describe adsorption on a heterogeneous surface. According to this model, since the adsorption centers have different energy values, the active sorption centers with maximum energy are filled first [51].

(9) $$lnq_{e}=lnk_{F}+\frac{1}{n}lnC_{e}$$

where $k_{F}$ is the Freundlich isotherm constant related to the adsorption capacity (μg/g), and n is the adsorption intensity indicating the extent of the adsorbent–adsorbate interaction.

**Figure 8:**
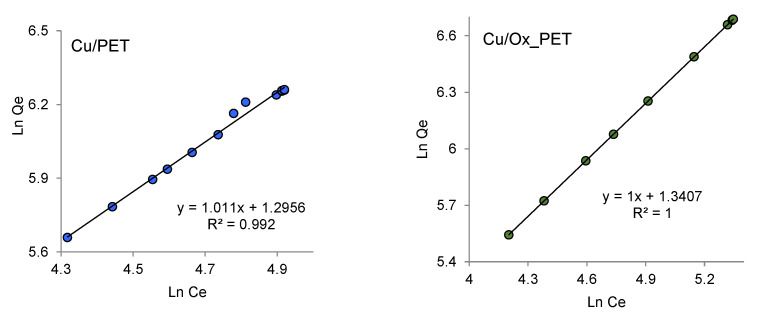
Freundlich adsorption isotherms of As(III) adsorption on composite TeMs.

Figure 8 shows the experimental data adapted to the linear Freundlich equation. The *n* constant is an empirical parameter related to the adsorption strength, which varies depending on the heterogeneity of the adsorbent. To facilitate adsorption, the value of *n* should be in the range of 1–10 [63]. The $k_{F}$ values of Cu/PET and Cu/Ox_PET were 3.65 and 3.82 μg/g, respectively, indicating that the adsorption capacity of the composites improved after chemical modification (i.e., oxidation) of the polymer template. This is consistent with the Langmuir isotherm. For both composites, the adsorption intensity (*n*) is equal to unity (Table 3), which indicates the possibility of As(III) adsorption on the surface of the composites. The regression coefficients (R^2^) are also equal to unity in Figure 8, indicating a very good agreement with the Freundlich adsorption isotherm. It is obvious that the Freundlich isotherm model describes the adsorption process better than the Langmuir model. This confirms the existence of a heterogeneous adsorption surface in which both the copper MTs and PET template are involved in the adsorption process.

#### 3.3.3. Dubinin–Radushkevich (DR) Isotherm

The DR isotherm model is often used for the description of adsorption, especially in microporous materials [64]. It is a semi-empirical equation suitable for adsorption following the pore-filling mechanism and assumes that adsorption has a multilayer nature involving Van der Waals forces. This isotherm is best suited for physical adsorption processes [59]. The linear form of the DR isotherm is defined by Equation (10) [65]:

(10) $$lnq_{e}=lnQ_{d}-\beta \varepsilon^{2}$$

$\mathrm{where}\;Q_{d}$ (μg/g) is the adsorption capacity of the DR monolayer, β (mol^2^/kJ^2^) is a constant associated with the free energy of sorption, and ε is the Polyanyi potential determined by the following relationship:

(11) $$\varepsilon =RTln\left(1+\frac{1}{C_{e}}\right)$$

**Figure 9:**
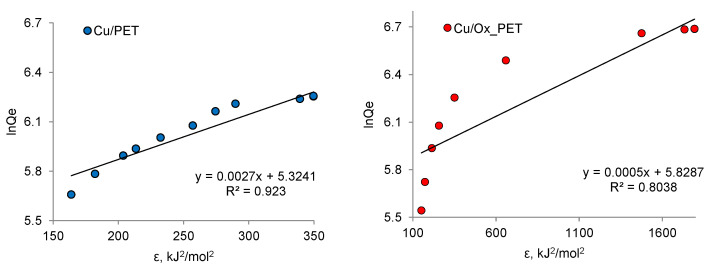
Dubinin–Radushkevich (DR) adsorption isotherms of As(III) adsorption on composite TeMs.

The β and $Q_{d}$ values were calculated from the slope and intersection, respectively (Figure 9), and the results are shown in Table 5. The average free energy of adsorption, $E_{DR}$, which characterizes the free energy of the system due to the transfer of one mole of ions from the solution to the solid surface, was calculated from the β values using the following equation:

(12) $$E_{DR}=\frac{1}{\sqrt{-2\beta }}$$

The importance of determining the value of $E_{DR}$ stems from the fact that its numerical value can be used to gain insight into the nature of the interactions between As(III) and active centers on the composite surface. Physical adsorption is considered to occur when the adsorption energy $E_{DR}$ is less than 8 kJ/mol. When $E_{DR}$ is between 8 and 16 kJ/mol, the process proceeds according to ion exchange theory, and chemisorption is observed at $E_{DR}$ values in the range of 20–40 kJ/mol [59]. The high $E_{DR}$ value observed for the Cu/Ox-PET composite indicates that the interaction between the adsorbate and the composite is based on chemisorption. However, very low R^2^ values indicate that the experimental data fit the DR adsorption isotherm model poorly, and therefore, the values obtained may fall outside the confidence interval. In other studies, for example, using magnetite NPs, the adsorption free energy was calculated as 7.6 kJ/mol, and the interactions between As(III) and the NPs were of physical type [66]. Similar results (0.84 kJ/mol) have been reported for the adsorption of arsenic by copper slug [67]. The sorption energy of the aluminum doped manganese copper ferrite/polymer composite prepared by chemical coprecipitation technique was found to be 40.98 kJ/mol, which confirms the chemisorption phenomenon [68]. A comparison of the adsorption capacities of different As(III) adsorbents with those attained in this study is presented in Table 6.

It should be noted that it is rather difficult to directly compare the data of various studies as some determining parameters, such as amount of loaded sorbent, agitation speed, pH value, and temperature of sorption, are not exactly the same. Still, it can easily be said that our results closely compete with the existing alternatives and that the obtained composite membranes are promising, particularly considering their practicality, ease of use, and low cost.

## 4. Conclusions

The present study suggests that PET track-etched membranes loaded with copper MTs have a very high potential for the removal of As(III) from aqueous medium. Kinetics and adsorption isotherms well explain the adsorption mechanisms for composite adsorbents prepared using an oxidized or pristine PET track-etched template. The adsorption of As(III) was rapid and reached equilibrium in ~6 h. The pseudo-second-order equation fitted well in both composites (i.e., Cu/PET and Cu/Ox_PET).

Three adsorption isotherm models were examined, and the relevant constants and parameters were determined. The analysis revealed a very good correlation between experimental data and the Freundlich isotherm, suggesting a multilayer adsorption on an energetically heterogeneous surface. The removal efficiency of As(III) is increased when an oxidized template is used. The amount of copper loaded to the template and the specific surface area of the composite was significantly higher when the track-etched PET template was oxidized. The oxidation process provides an increase in surface porosity too, which positively contributes to the adsorption process. As a result of these effects, and because the size of copper crystallites is smaller when an oxidized template is used, arsenic absorption was found to be higher for Cu/Ox_PET. We, therefore, recommend oxidation as a step that can be considered in the preparation of similar membranes to achieve higher removal efficiencies. Given the high adsorption capacity and ease of use of the composite adsorbent, the prepared samples offer a promising alternative for the removal of As(III) from the aqueous medium.

## Figures and Tables

**Table 1:** Structural properties of the composite track-etched membranes (TeMs).

Composite	Structural Parameters of Embedded MTs, nm		Specific Surface Area, m^2^/g	Deposition Rate, R, mg/(cm^2^ h)	Amount of Cu Loaded, mg/cm^2^
	Wall Thickness	Inner Diameter			
Cu/PET	73.7 ± 8.5	269.0 ± 10.5	11.4 ± 1.7	5.52	0.77 ± 0.03
Cu/Ox_PET	64.0 ± 6.5	288.2 ± 13.8	14.5 ± 2.1	5.45	0.82 ± 0.01

**Table 2:** Crystal structure of the composites according to XRD data.

Composite	Phase	Symmetry Group	hkl *^a^*	2*θ*º	*d ^b^* Å	*L ^c^* nm	*a ^d^* Å	DC *^e^* %	FWHM *^f^*	Phase Ratio %
Cu/PET	Cu	Fm3m (225)	111	43.46	2.081	18.0 ± 2.7	3.602	43.8	0.455	100
			200	50.60	1.803				0.626	
			220	74.29	1.276				0.557	
			311	90.06	1.089				0.788	
			222	-	-				-	
Cu/Ox_PET	Cu	Fm3m (225)	111	43.55	2.076	15.0 ± 2.8	3.604	52.5	0.401	100
			200	50.70	1.799				0.553	
			220	74.37	1.275				0.537	
			311	90.13	1.088				0.704	
			222	-	-				-	

*^a^* Miller indices for corresponding planes, *^b^* spacing between planes, *^c^* average crystallite size, *^d^* crystal lattice parameter, *^e^* degree of crystallinity, *^f^* full width at half maximum.

**Table 3:** Comparison of the pseudo-first- and pseudo-second-order kinetic parameters and Elovich α and β values of As(III) sorption by Cu/PET and Cu/Ox_PET.

Composite Adsorbent		Pseudo-First-Order Model			Pseudo-Second-Order Model			Elovich Model		
		$k_{1},$ min^−1^	$q_{e},$ mg/g	R^2^	$k_{2}\times 10^{-4},$ g/mg×min	$q_{e,}$ mg/g	R^2^	*α*, mg/g×min	*β*, mg/min	R^2^
Cu/PET		0.012	0.283	0.982	0.74	0.55	0.999	1.45	0.014	0.979
Cu/Ox_PET		0.012	1.942	0.897	0.07	1.0	0.995	3.95	0.005	0.941

**Table 4:** Langmuir and Freundlich isotherm constants for the adsorption of As(III) onto composite TeMs.

Sorbent	Langmuir Isotherm			Freundlich Isotherm		
	$Q_{0}$, μg/g	*b*, L/μg	R^2^	$k_{F},$ μg/g	n	R^2^
Cu/PET	158.7	0.027	0.97	3.65	0.99	0.99
Cu/Ox_PET	238.1	0.118	0.92	3.82	1.0	1.0

**Table 5:** DR isotherm constants for the adsorption of As(III) on composite TeMs.

Sorbent	$Q_{d},$ μg/g	β, mol^2^/kJ^2^	$E_{DR},$ kJ/mol	R^2^
Cu/PET	217.0	0.002	17.2	0.87
Cu/Ox_PET	379.9	0.001	31.6	0.80

**Table 6:** Comparison of the As(III) adsorption capacity of the composite TeMs loaded with copper MTs and other sorbents.

Adsorbent	Sorption Conditions		*Q*e, mg/g	Ref.
	Initial Concentration of Adsorbate, ppm	Amount of Adsorbent Utilized, mg		
CuO nanoparticles (NPs)	100.0	2000.0	26.90	[69]
Fe/Cu NPs	5.0	10.0	19.70	[70]
TiO_2_ NPs embedded in the chitin hydrogel	0.1	200.0	3.10	[71]
Copper oxide incorporated mesoporous alumina	1.0	200.0	2.16	[72]
Fe_3_O_4_ NPs	0.13	10.0	1.38	[66]
Aluminum doped manganese copper ferrite/polymer composite	0.2	50.0	0.05	[68]
Granular Ce–Ti sorbent	5.0	10.0	0.90	[73]
Activated alumina	1.0	1000.0	0.08	[74]
Cu/PET composite TeMs	50.0	3.8	0.52	This study
Cu/Ox_PET composite TeMs	50.0	4.0	0.80	

## Supplementary Materials

The following are available online at https://www.mdpi.com/2077-0375/11/2/116/s1: Figure S1. XPS high resolution C1s spectra of the “etched-only” (a) and oxidized (b) polyethylene terephthalate (PET) template (b), O1s spectra of the “etched-only” (c) and oxidized (d) PET template; Figure S2. Fourier-transform infrared spectroscopy (FTIR) spectra of “etched-only” and oxidized PET templates; Figure S3. An Energy-dispersive X-ray spectroscopy (EDX) spectra with percentage of elements (at.%) for a Cu/PET and Cu/Ox_PET composite track-etched membranes.

## Abbreviations

TeM	track-etched membrane
PET	polyethylene terephthalate
XRD	X-ray diffraction
Cu/PET	composite membrane synthesized using an “etched-only” template
Cu/Ox_PET	composite membrane synthesized using an oxidized template
MT	microtube
SEM	scanning electron microscopy
XPS	X-ray photoelectron spectroscopy
EDX	energy dispersive X-ray analysis
Qe	amount of As(III) adsorbed by the unit mass of copper (mg/g)
C_0_	feed As(III) concentration (mg/L)
C_e_	concentration of As(III) in aliquots (mg/L)
DC	degree of crystallinity (%)
L	average crystallite size (nm)
$q_{t}$	adsorption capacity at time t (mg/g)
$k_{1}$	First-order reaction rate constant (min^−1^)
$k_{2}$	pseudo-second-order rate constant of adsorption ($\frac{g}{\mathrm{mg}\times \min}$)
α	initial rate of the adsorption process, mg/g × min
β	desorption constant (g·mmol^−1^)
Ra	roughness of the composite (nm)
b	constant related to the energy of adsorption (i.e., Langmuir constant (L/μg))
Ce	equilibrium concentration of adsorbate (mg/L)
$Q_{0}$	maximum monolayer coverage capacity (mg/g)
$k_{F}$	Freundlich isotherm constant related to the adsorption capacity (μg/g)
$Q_{d}$	adsorption capacity of the Dubinin–Radushkevich monolayer (μg/g)
β	constant associated with the free energy of sorption (mol^2^/kJ^2^)
$E_{\mathrm{DR}}$	free energy of adsorption (kJ/mol)
