# Supplementary material for: Kinetic and Isotherm Study of As(III) Removal from Aqueous Solution by PET Track-Etched Membranes Loaded with Copper Microtubes
Source: Membranes (Basel). 2021 Feb 6;11(2):116. doi: 10.3390/membranes11020116 (PMC7914724; doi:10.3390/membranes11020116)
Supplement: Supplementary file 1 [file membranes-11-00116-s001.pdf]

membranes

MDPI

Supplementary information

Kinetic and Isotherm Study of As(III) Removal From Aqueous Solution By PET Track-Etched Membranes Loaded with Copper Microtubes

Alyona V. Russakova [1], Liliya Sh. Altynbaeva [2,3], Murat Barsbay [4], Dmitriy A. Zheltov [2], Maxim V. Zdorovets [2,5,6] and Anastassiya A. Mashentseva [2,3,*]

[1] The School of Information Technologies and Intelligent Systems, D.Serikbayev East Kazakhstan State Technical University, 070004 Ust-Kamenogorsk, Kazakhstan; Arussakova@gmail.com
[2] The Institute of Nuclear Physics of the Republic of Kazakhstan, 050032 Almaty, Kazakhstan; lilija310378@gmail.com (L.S.A.); zheltovda@gmail.com (D.A.Z.); mzdorovets@gmail.com (M.V.Z.)
[3] Department of Chemistry, L.N. Gumilyov Eurasian National University, 010008 Nur-Sultan, Kazakhstan
[4] Department of Chemistry, Hacettepe University, 06800 Ankara, Turkey; mbarsbay@hacettepe.edu.tr
[5] Department of Intelligent Information Technologies, The Ural Federal University, 620002 Yekaterinburg, Russia
[6] Engineering Profile Laboratory, L.N. Gumilyov Eurasian National University, 010008 Nur-Sultan, Kazakhstan
[*] Correspondence: a.mashentseva@inp.kz; Tel.: +7-707-322-4399

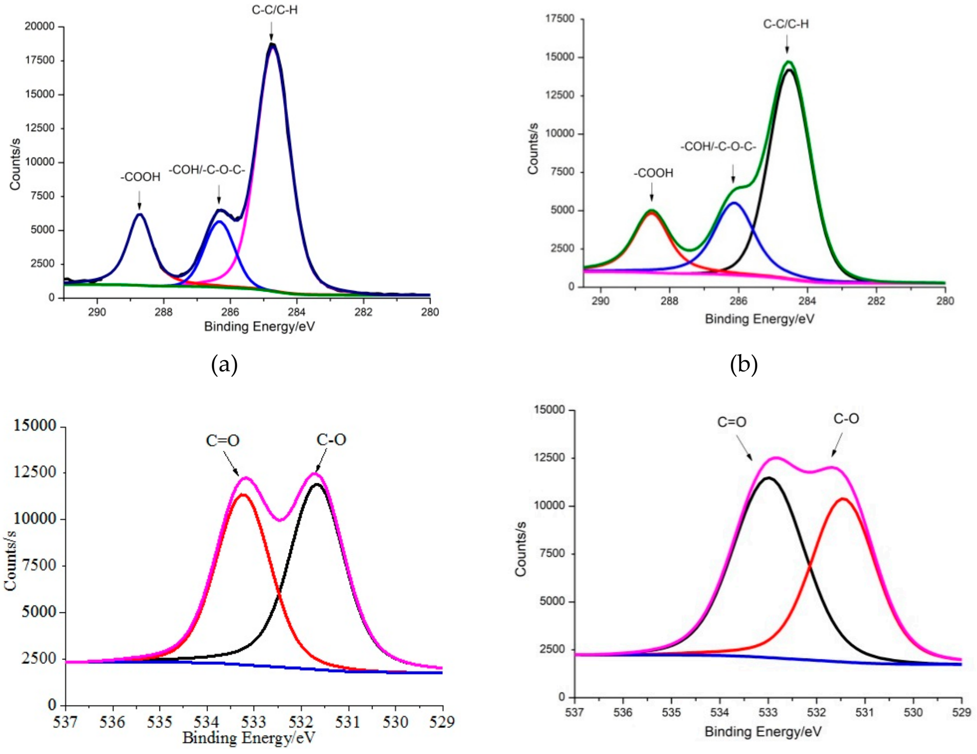

(a) (b)

(c) (d)

Figure S1. XPS high resolution C1s spectra of the "etched-only" (**a**) and oxidized (**b**) polyethylene terephthalate (PET) template (b), O1s spectra of the "etched-only" (**c**) and oxidized (**d**) PET template.

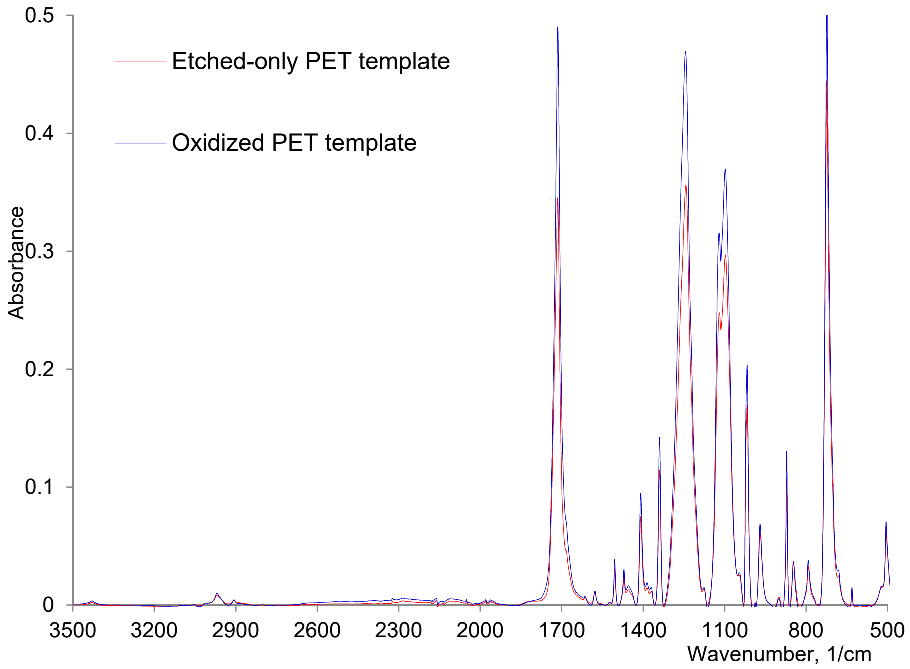

Figure S2. Fourier-transform infrared spectroscopy (FTIR) spectra of "etched-only" and oxidized PET templates.

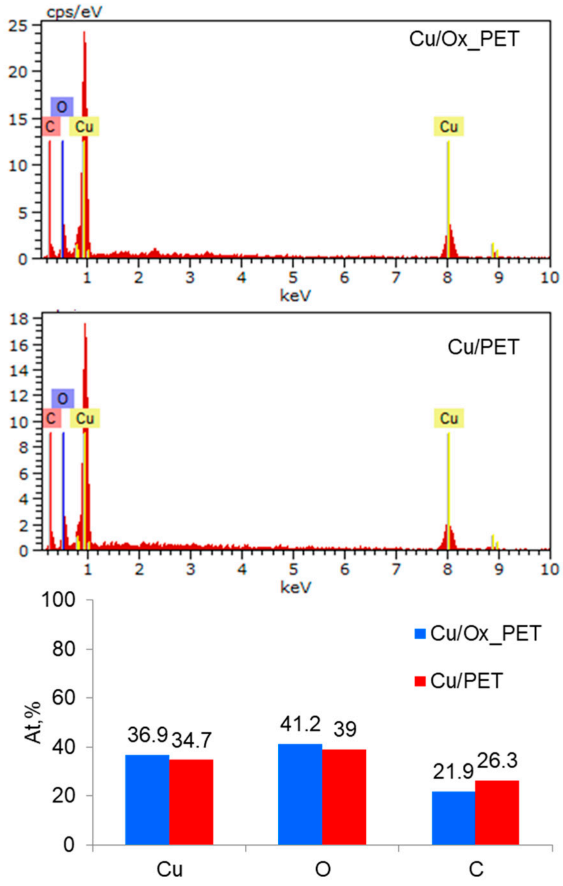

Figure S3. An Energy-dispersive X-ray spectroscopy (EDX) spectra with percentage of elements (at.%) for a Cu/PET and Cu/Ox_PET composite track-etched membrane.
